# Diagnostic accuracy of physical examination for detecting pelvic fractures among blunt trauma patients: a systematic review and meta-analysis

**DOI:** 10.1186/s13017-020-00334-z

**Published:** 2020-10-02

**Authors:** Yohei Okada, Norihiro Nishioka, Shigeru Ohtsuru, Yasushi Tsujimoto

**Affiliations:** 1 Department of Primary Care and Emergency Medicine, Graduate School of Medicine, Kyoto University, Shogoin Kawaramachi 54, Sakyo, Kyoto, 606-8507 Japan; 2 Department of Preventive Services, School of Public Health, Kyoto University, Kyoto, Japan; 3 Department of Healthcare Epidemiology, School of Public Health in the Graduate School of Medicine, Kyoto University, Kyoto, Japan; 4 Department of Nephrology and Dialysis, Kyoritsu Hospital, Osaka, Japan

**Keywords:** Pelvic fractures, Physical examination, Trauma, Diagnostic accuracy, Imaging, Decision curve analysis, Net-benefit

## Abstract

**Background:**

Pelvic fractures are common among blunt trauma patients, and timely and accurate diagnosis can improve patient outcomes. However, it remains unclear whether physical examinations are sufficient in this context. This study aims to perform a systematic review and meta-analysis of studies on the diagnostic accuracy and clinical utility of physical examination for pelvic fracture among blunt trauma patients.

**Methods:**

Studies were identified using the MEDLINE, EMBASE, and CENTRAL databases starting from the creation of the database to January 2020. A total of 20 studies (49,043 patients with 8300 cases [16.9%] of pelvic fracture) were included in the quality assessment and meta-analysis. Two investigators extracted the data and evaluated the risk of bias in each study. The meta-analysis involved a hierarchical summary receiver operating curve (ROC) model to calculate the diagnostic accuracy of the physical exam. Subgroup analysis assessed the extent of between-study heterogeneity. Clinical utility was assessed using decision curve analysis.

**Results:**

The median prevalence of pelvic fracture was 10.5% (interquartile range, 5.1–16.5). The pooled sensitivity (and corresponding 95% confidence interval) of the hierarchical summary ROC parameters was 0.859 (0.761–0.952) at a given specificity of 0.920, which was the median value among the included studies. Subgroup analysis revealed that the pooled sensitivity among patients with a Glasgow Coma Scale score ≥ 13 was 0.933 (0.847–0.998) at a given specificity of 0.920. The corresponding value for patients with scores ≤ 13 was 0.761 (0.560–0.932). For threshold probability < 0.01 with 10–15% prevalence, the net benefit of imaging tests was higher than that of physical examination.

**Conclusion:**

Imaging tests should be performed in all trauma patients regardless of findings from physical examination or patients’ levels of consciousness. However, the clinical role of physical examination should be considered given the prevalence and threshold probability in each setting.

## Introduction

Pelvic fracture can cause retroperitoneum hemorrhage and hemorrhagic shock among blunt trauma patients [1–4]. It is estimated that 10–15% of patients with pelvic fractures are in shock when they present at an emergency department and have a mortality rate of approximately 30% [1]. Therefore, early diagnosis and treatment with retroperitoneum packing or trans-arterial embolization are essential for good outcomes [2, 3].

Pelvic fracture diagnosis entails physical examination of the pelvis, which is generally performed in prehospital settings or at an emergency department [4–6]. It includes the inspection of deformities and the palpation of the pelvis to assess stability; it can be useful as a triage tool or to reduce the frequency of the imaging test [7–9]. Nevertheless, some studies [10–12] have challenged the reliability of physical examination, in particular, among patients with impaired consciousness. A false-negative (FN) result in this context may delay treatment, thus increasing mortality risk [13]. Given these considerations, some institutions perform computed tomography (CT) scans for all trauma patients regardless of physical examination findings [14, 15]. Although potentially useful, CT scans increases the exposure to radiation and the medical costs.

To understand the clinical role of physical examination in this context, it is necessary to consider its diagnostic ability and clinical utility. However, few systematic reviews and meta-analyses have been performed to estimate these parameters while adhering to methodological guidelines [16–18]. This study performed a systematic review and meta-analysis to assess the diagnostic accuracy and clinical utility of physical examination for pelvic fracture among blunt trauma patients.

## Methods

We performed a systematic review and meta-analysis of studies on diagnostic test accuracy (DTA). We adhered to the methodological standards outlined in the *Handbook for DTA Reviews* of Cochrane [16] and used the Preferred Reporting Items for a Systematic Review and Meta-analysis of Diagnostic Test Accuracy Studies (i.e., PRISMA-DTA) [18] in reporting our findings. The review protocol is available on a preprint server (medRexiv) [19] and was prospectively registered with the University Hospital Medical Information Network Clinical Trials Registry (UMIN000038785) [20, 21].

### Population, index test, and target condition

The target participants were blunt trauma patients with potential pelvic injury. The index test of interest was physical examination for pelvic fracture, which is defined as follows [4, 5, 7, 9]: inspection: presence of pelvic deformity, hip dislocation, ecchymosis, laceration, hematoma over the pelvic ring; palpation: pelvic bone pain or tenderness, instability or abnormal movement in applying manual internal and external rotational stress, and anteroposterior and superior–inferior stress. In addition, we considered the definitions used in primary studies. However, studies with discrepant definitions of index test positive were excluded from the sensitivity analysis. The target condition was defined as pelvic fracture due to blunt trauma diagnosed by x-ray or CT scan by an emergency physician, trauma surgeon, or orthopedic or radiology specialist, alongside the criteria defined by primary study authors.

### Ethics approval and consent to participate

The need for ethical approval and consent was waived for this systematic review.

### Study eligibility and selection

We included all studies on the diagnostic accuracy of physical examinations for detecting pelvic fractures in blunt trauma patients treated in any setting. All study designs were eligible, including prospective, retrospective, and observational (cohort or cross-sectional) studies and secondary analyses of randomized controlled trial data. We excluded diagnostic case-control studies (two-gate study) and case studies that lacked DTA data, namely true-positive (TP), false-positive (FP), true-negative (TN), and FN values.

Two authors independently screened each study for eligibility and extracted the data. Disagreements among reviewers were resolved via discussions or by the third reviewer. Excluded studies (with reasons) are listed in the supplementary file (S-Table 1).

### Electronic searches

To identify all eligible studies, we searched the Medical Literature Analysis and Retrieval System Online (MEDLINE) via Ovid (accessed on January 10, 2020), the Excerpta Medica Database (EMBASE) (accessed on January 9, 2020), and the Cochrane Central Register of Controlled Trials (CENTRAL) (accessed on January 14, 2020). We also searched the International Clinical Trials Registry Platform and ClinicalTrials.gov (accessed on January 14, 2020) for ongoing and unpublished studies. There were no restrictions on language or publication date for this review. The reference lists of eligible studies were searched manually for other potentially relevant studies, and the details of the search strategy are described in a supplementary file (S-Method).

### Data extraction and quality assessment

The following data were extracted: study characteristics (author, year of publication, country, design, sample size, clinical settings, conflict of interest, and funding source), patient characteristics (inclusion/exclusion criteria and patient clinical and demographic characteristics), index test (setting, method, and performer of the physical examination), reference standard (modality and its interpreter), and diagnostic accuracy parameters (TP, FP, FN, and TN).

Two investigators evaluated the risk of bias by using the QUADAS-2 tool [17], which includes four risk of bias domains and three domains of applicability. Any disagreements were resolved via discussions or by the third reviewer. Assessment findings were presented using the traffic light plot and weighted summary plot “robvis” in R package [22]. Given the absence of evidence for publication bias in DTA studies and the lack of reliable methods for its assessment, no statistical evaluation of publication bias was performed [16].

### Statistical analysis and data synthesis

The *Cochrane Handbook for Systematic Reviews of Diagnostic Test Accuracy* methodology was applied [16]. Study diagnostic sensitivity and specificity estimates with 95% confidence intervals (CIs) for physical examination were captured in paired forest plots to inspect the between-study variance. Although we had planned to use a bivariate random-effects model for the meta-analysis, the between-study heterogeneity was high, thus precluding accurate summary estimation. As a result, we used a summary receiver operating curve (ROC) fitted as a hierarchical summary ROC (HSROC) nonlinear mixed model [23]. This approach allows the incorporation of data at different thresholds or from different physical examination procedures. By using HSROC parameter estimates, we fixed specificity at the median value of all included studies; we then calculated the sensitivity with 95% CIs in the same manner as the previous Cochrane review [24]. Given that DTA studies typically contain fewer patients with the target condition than patients without the target condition, sensitivity estimates are often made with less certainty than estimates of specificity [16].

### Assessment of clinical utility

For clinical decision-making, we calculated the estimates of TP, TN, FP, and FN per 1000 patients with a 5%, 10%, or 15% prevalence of pelvic fracture by using the pooled diagnostic accuracy [25–28]. Moreover, we calculated the net benefit and performed decision curve analysis [29, 30]. Net benefit refers to the difference between the benefit and weighted harm of the test calculated as [proportion of TP − proportion of FP × weighting]. Weighting is calculated by threshold probability (*p*) as [*p* / (1 − *p*)] and refers to the number of FP patients who have clinical importance equal to one TP patient. Threshold probability refers to the level of diagnostic certainty above which the patient would be treated on the basis of hospital policy or their own preference. For example, if “*p* = 0.1,” the weighting is 0.1 / (1 − 0.1) = 1/9 (i.e., 9 FP is equal to 1 TP). Therefore, if 10% of patients are TP (9 FP and 1 TP), all patients should be treated. In general, for decision curve analysis, net benefit is plotted using index test findings under several thresholds of probability. Furthermore, net benefit is plotted if all patients are treated as positive or negative regardless of the index test result. Decision curve analysis can help obtain the highest net benefit. In the current study, we assumed that all patients were positive and that an imaging test was performed or all patients were negative and that no further imaging tests were performed regardless of the physical examination findings. Finally, we compared the net benefit values to assess when physical examination was useful.

### Investigations of heterogeneity

The parameters for subgroup analysis were as follows: age (adult/children), patient condition, setting, diagnosing clinician, pelvic fractures, and reference standard modality. We used a paired forest plot for subgroup analysis and performed meta-regression with subgroups as covariates. We plotted the HSROC parameters and calculated the pooled diagnostic ability (sensitivity, specificity, and diagnostic odds ratio [DOR]) and relative DOR (RDOR) with 95% CIs. Furthermore, we assessed the significance of the differences between the test results by using the likelihood ratio test. Subgroup analysis results were used in the decision curve analysis as described.

### Sensitivity analysis

We assessed the robustness of the results by excluding studies with discrepant definitions of index test positive or reference. Furthermore, we performed post-hoc sensitivity analysis to exclude studies with high risk of bias in at least one domain.

All analyses were performed using SAS studio (SAS Institute Inc. Cary, NC, USA) with the “MetaDAS” statistical package [31], Review Manager 5.3 (Cochrane Collaboration, London, UK), and CAST-HSROC [32]. All statistical analyses were conducted with a two-sided alpha error of 5%.

## Results

**Fig. 1:**
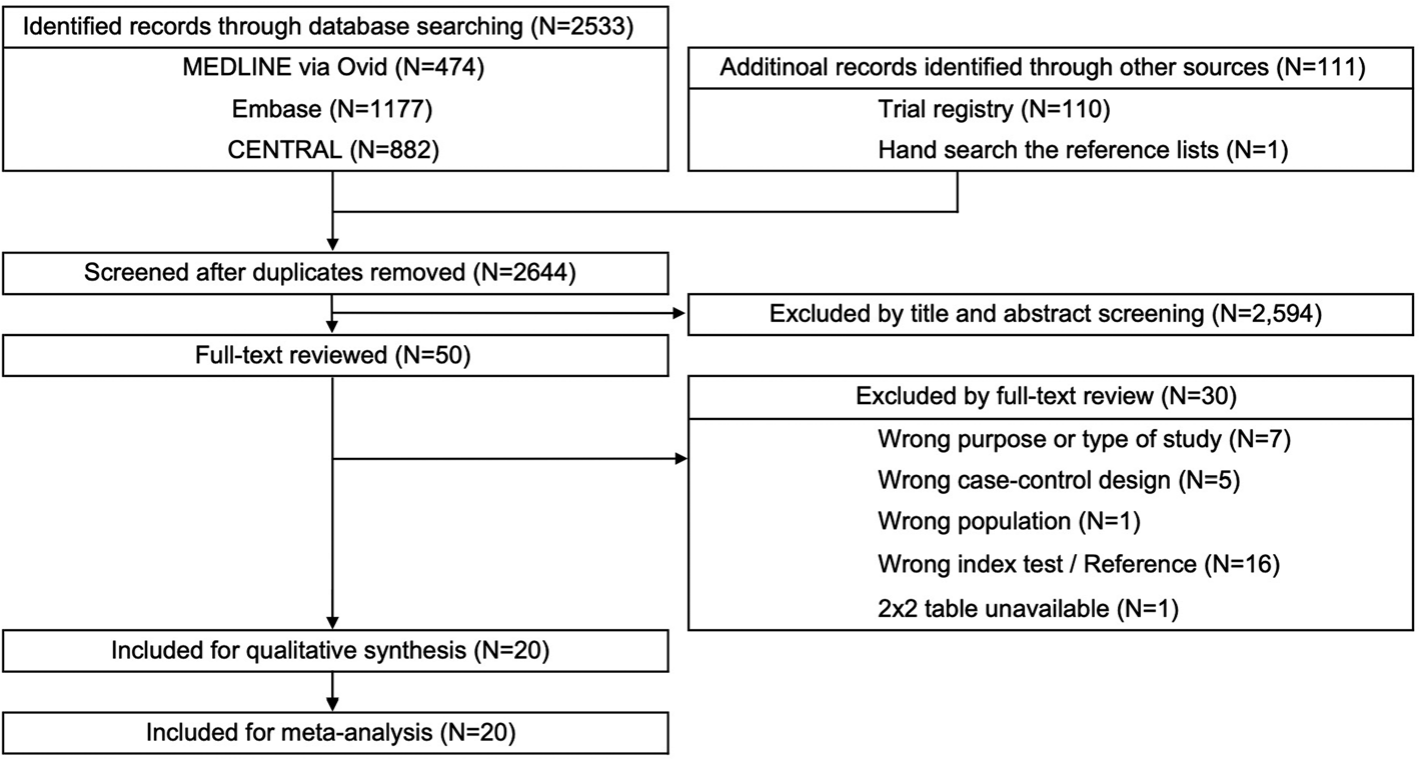
Study flowchart (PRISMA flowchart)

A total of 2644 studies were screened. Twenty studies met the eligibility criteria and were included [10–12, 33–49] in the quality assessment and meta-analysis (Fig. 1 and S-Table 1 in supplementary file).

### Study characteristics

Data from 49,043 patients, including 8300 patients (16.9%) with pelvic fracture, were included in the analysis (Table 1). The median prevalence of pelvic fracture was 10.5% (IQR: 5.1–16.5). Sixteen studies [10, 12, 33–36, 38–44, 47–49] were prospective, and four studies [11, 37, 45, 46] were retrospective. Most studies were set at a trauma center or emergency department of a university hospital. Three studies [37, 39, 46] included children (< 18 years). Seven studies [33, 35, 36, 38, 41, 44, 49] included patients who were either alert or had minor impairments to consciousness (Glasgow Coma Scale [GCS] score ≥ 13); other studies included patients with GCS score ≤ 13. Patient characteristics, index test definitions, and reference standards used in each study are summarized in Tables 2 and 3. Physical examination included inquiries about pelvic pain, inspection and palpation of the pelvis, assessment of the stability of the pelvis, and other procedures. Physical examinations were performed by an emergency physician in a trauma bay, emergency department, or surgical department. The reference standards were x-ray [33–36, 38, 40, 44, 46, 49], unclear [11], or x-ray or CT [10, 12, 37, 39, 41–43, 45, 47, 48]. Findings were interpreted by a radiologist [33–35, 39, 41, 43, 45, 46, 49], surgeon [12, 33, 35, 36, 42], or an unreported specialist [10, 11, 37, 38, 40, 44, 47, 48]. One study [48] focused on an unstable pelvic fracture.

**Table 1:** Summary of primary study characteristics

Author	Year	Country	Design	Setting	*N*	Pelvic Fx prevalence	Fund	COI
Civil et al. [33]	1988	USA	Pro	Trauma C	133	8 (6.0%)	Unclear	Unclear
Grant [34]	1990	UK	Pro	ED	36	22 (61.1%)	Unclear	Unclear
Salvino et al. [35]	1992	USA	Pro	Trauma C	810	39 (4.8%)	Unclear	Unclear
Yugueros et al. [36]	1995	USA	Pro	ED	608	59 (9.7%)	Unclear	Unclear
SD. John et al. [37]	1996	USA	Retro	Pediatric ED	292	6 (2.1%)	Unclear	Unclear
Heath et al. [38]	1997	USA	Pro	ED	82	9 (11%)	Unclear	Unclear
Junkins et al. [39]	2001	USA	Pro	Pediatric Trauma C	140	16 (11.4%)	Unclear	Unclear
Duane et al. [40]	2002	USA	Pro	Trauma C	247	45 (18.2%)	Unclear	Unclear
Gonzalez et al. [41]	2002	USA	Pro	Trauma C	2176	97 (4.5%)	Unclear	Unclear
Pehle et al. [42]	2003	Germany	Pro	ED	979	111 (11.3%)	Unclear	Unclear
Waydhas et al. [12]	2007	Germany	Pro	Trauma C	784	93 (11.9%)	Unclear	Unclear
Duane et al. [43]	2008	USA	Pro	Trauma C	1388	168 (12.1%)	Unclear	Unclear
Duane et al. [44]	2009	USA	Pro	Trauma C	197	8 (4.1%)	Unclear	Unclear
Shlamovitz et al. [45]	2009	USA	Retro	Trauma C	1316	109 (8.3%)	Unclear	Unclear
Lagisetty et al. [46]	2012	USA	Retro	Pediatric Trauma C	504	19 (3.8%)	Unclear	Unclear
Lustenberger et al. [11]	2016	Germany	Retro	Trauma registry	35490	7201 (20.3%)	Unclear	Unclear
Majidinejad et al. [47]	2018	Iran	Pro	ED	3527	224 (6.4%)	Declared	Declared
Schweigkofler et al. [48]	2017	Germany	Pro	Trauma C	147	57 (38.8%)	Unclear	Declared
Leent et al. [10]	2019	Netherlands	Pro	Trauma C	54	11 (20.3%)	Unclear	Unclear
Moosa et al. [49]	2019	Pakistan	Pro	ED	133	16 (12.0%)	Declared	Declared

*Pro* prospective study, *Retro* retrospective study, *Trauma* C trauma center, *ED* emergency department, *N* the number of total patients included in analysis, *Fx* fracture, *COI* conflict of interest

**Table 2:** Summary of primary study characteristics, continued

Author	Year	Inclusion		Index test							Reference standard	
		Age (year)	GCS	Setting	Ask	Inspection	Palpation	Stability	Other	Modality	Radiologist	Blind
Civil et al. [33]	1988	–	15	In-hos	+	+	+	+	+	Xp	+	?
Grant [34]	1990	–	–	In-hos	−	−	−	+	−	Xp	+	?
Salvino et al. [35]	1992	≥ 12	≥ 13	In-hos	+	+	+	+	+	Xp	+	−
Yugueros et al. [36]	1995	> 13	≥ 14	In-hos	−	−	−	+	−	Xp	−	+
SD. John et al. [37]	1996	< 18	–	In-hos	+	?	?	?	?	Xp or CT	−	?
Heath et al. [38]	1997	≥ 18	≥ 14	In-hos	?	?	?	?	?	Xp	−	?
Junkins et al. [39]	2001	< 18	–	In-hos	−	+	+	+	+	Xp or CT	+	?
Duane et al. [40]	2002	–	–	In-hos	+	+	+	+	−	Xp	?	?
Gonzalez et al. [41]	2002	> 14	≥14	In-hos	+	+	−	+	+	Xp or CT	+	+
Pehle et al. [42]	2003	–	–	In-hos	−	+	+	+	+	Xp or CT	−	?
Waydhas et al. [12]	2007	–	≤13	In-hos	−	−	−	+	−	Xp or CT	−	+
Duane et al. [43]	2008	> 16	–	In-hos	+	+	−	+	−	Xp or CT	+	?
Duane et al. [44]	2009	> 16	≥ 13	In-hos	+	+	−	+	+	Xp	?	?
Shlamovitz, et al. [45]	2009	–	–	In-hos	+	+	+	+	−	Xp or CT	+	?
Lagisetty et al. [46]	2012	< 18	–	In-hos	+	+	+	+	−	Xp	+	+
Lustenberger et al. [11]	2016	–	–	Pre-hos	?	?	?	?	?	?	?	?
Majidinejad et al. [47]	2018	5–64	–	In-hos	+	−	+	−	−	Xp or CT	?	?
Schweigkofler et al. [48]	2017	–	–	Both	?	?	?	?	?	Xp or CT	?	?
Leent et al. [10]	2019	≥ 18	–	Pre-hos	−	−	−	+	−	Xp or CT	?	?
Moosa et al. [49]	2019	≥ 16	15	In-hos	−	−	+	−	−	Xp	+	?

*GCS* Glasgow coma scale, *In*-*hos* In-hospital, *Pre*-*hos* Pre-hospital, *Xp* X-ray picture, *CT* computed tomography, ? unclear

**Table 3:** Demographic and clinical characteristics of patients included in the primary studies

Author	Year	Age (years)	Men	Mechanism	GCS	Severity
Civil et al. [33]	1988	PE+31/ PE- 34	–	Fx+: MVA100%	15: 100%	ISS PE+11.7/PE- 8.6
Grant [34]	1990	46, range (9–95)	47%	TA 61%	≥ 13: 94%	TS 15–16: 92%
Salvino et al. [35]	1992	33, range (12–78)	66%	MVA 58%	–	ISS 11
Yugueros et al. [36]	1995	Median 33, range (14–90)	73%	–	15: 74%	Median ISS 8
SD. John et al. [37]	1996	10, range (5 m– 17)	55%	MVA 55%	–	–
Heath et al. [38]	1997	Range (18– 81)	–	MVA 79%	≥14:100%	–
Junkins et al. [39]	2001	Fx+9.8/Fx-7.8	51%	MVA Fx+:69%/ Fx-: 54%	–	Median ISS Fx+:9/ Fx-: 8
Duane et al. [40]	2002	Fx+:3 6(17)/ Fx-:34 (19)	–	–	Fx+ 14.4 (2.1)/ Fx- 14.4 (2.1)	ISS Fx+:11.5 (7.4)/ Fx-:5.9 (6.6)
Gonzalez et al. [41]	2002	36, range (14–93)	62%	MVA73%	≥ 14:100%	–
Pehle et al. [42]	2003	PE+: 40 (22)/ PE-: 44 (20)	71%	–	PE+10.7 (4.8)/ PE-:10.8 (4.6)	ISS PE+42.3 (19.6)/ PE- 19.9 (15.6)
Waydhas et al. [12]	2007	–	71%	–	9.8 (4.7)	ISS 23.3 (17.4)
Duane et al. [43]	2008	Fx+: 41 (18)/ Fx-: 39 (17)	–	–	Fx+: 12.3 (4.6)/Fx-: 13.9 (3.1)	–
Duane et al. [44]	2009	34 (12)	80%	MVA 76%	12.8 (4.1)	–
Shlamovitz et al. [45]	2009	36 (20)	68%	MVA 44%	14 (2.6)	RTS 10.6 (1.5)
Lagisetty et al. [46]	2012	–	–	MVA68%	< 14: 11.3%	–
Lustenberger et al. [11]	2016	43 (20) in true-positive	66% in TP	–	11.8 (4.4) in TP	29.6 (14.6) in TP
Majidinejad et al. [47]	2018	32 (14)	76%	–	–	–
Schweigkofler et al. [48]	2017	46	69%	TA 51%	–	–
Leent et al. [10]	2019	49(20)	70%	TA 63%	≤13: 46%	–
Moosa et al. [49]	2019	37(14)	92%	–	–	–

*GCS* Glasgow coma scale, *Fx* pelvic fracture, *PE* physical examination, *MVA* motor vehicle accident, *TA* traffic accident, *TS* trauma score, *ISS* injury severity score, *RTS* revised trauma score. Data is described as mean (standard deviation) or proportion (%) unless otherwise noted

### Risk of bias assessment

**Fig. 2:**
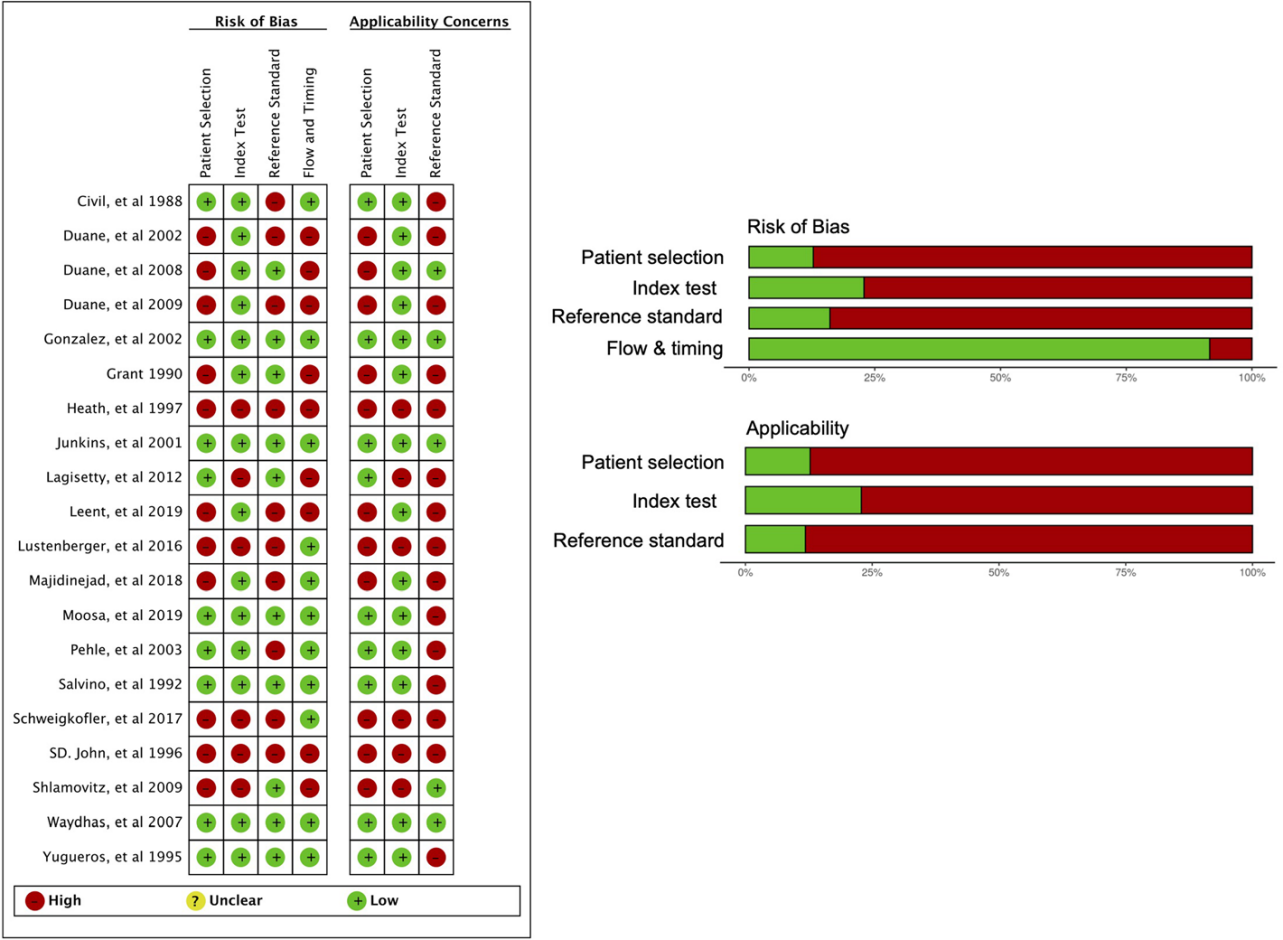
Summary of risk of bias assessment (QUADAS-2 tool). Green: low risk of bias or low concern in applicability. Red: high risk of bias or high concern in applicability. The assessment is weighted based on the sample size in each study in weighted summary plot. The detail of the assessment is described in supplementary file (S-Table 2)

For patient selection, we evaluated 11 studies [10, 11, 34, 37, 38, 40, 43–45, 47, 48] as having high risk or high concern in applicability (Fig. 2) because of poorly described inclusion criteria, nonreproducible methodology, inappropriate patient selection, or poor exclusion criteria, such as the selective exclusion of patients who did not have a reference standard (x-ray or CT) or complete physical examination data. In two studies [10, 11], it was not clear when the physical examination was performed. For the index test, we evaluated six studies [11, 37, 38, 45, 46, 48] as having high risk or high concern because the physical examination findings were retrospectively collected or because the index test was poorly described. For the reference standard, we evaluated nine studies [12, 34–36, 39, 41, 43, 45, 46, 49] as having low risk of bias because the readers of the imaging scans were blinded to the physical examination findings or because the reference was based on the radiologist’s findings; otherwise, studies were considered to have high risk of bias and high concern in applicability. Moreover, we evaluated five studies [34–36, 46, 49] as having high concern in applicability because of the reference standard being x-ray only despite CT scan being the current gold standard in trauma diagnosis. In patient flow assessment, we deemed nine studies [10, 34, 37, 38, 40, 43–46] to have high risk of bias because these studies excluded a certain number of patients from analysis without proper reporting. The overall quality of the included studies was low. The details of the assessment are shown in the supplementary file (S-Table 2).

### Results of meta-analysis

**Fig. 3:**
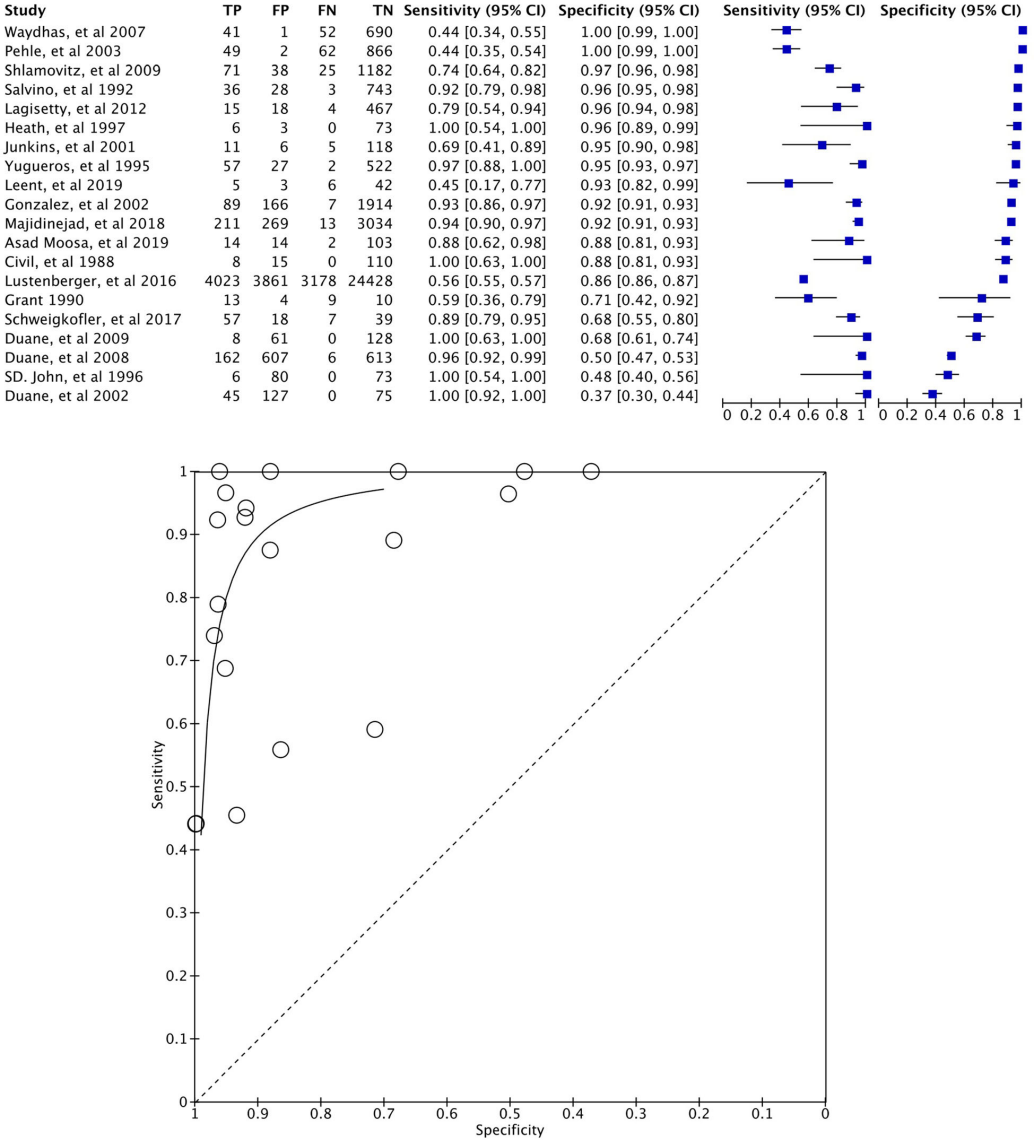
Paired forest plot and HSROC in primary analysis

The summary of the diagnostic accuracy and hierarchical ROC of physical examination for each study is presented in Fig. 3. DOR was 76.8 (95% CI 37.3–157.9). The calculated pooled sensitivity using HSROC parameters was 0.859 (95% CI 0.761–0.952) at a given specificity of 0.920 (median value among included studies). The positive and negative likelihood ratios were 10.7 (95% CI 9.5–11.9) and 0.153 (95% CI 0.05–0.26), respectively. Given a sensitivity of 0.859, the pooled specificity was 0.923 (95% CI 0.839–0.988). In a population of 1000 patients with a given pelvic fracture prevalence of 10%, the following was detected: 86 patients (95% CI 76–95) with true TP, 14 patients (95% CI 5–24) with FN, 831 patients (95% CI 755–889) with TN, and 69 patients (95% CI 11–145) with FP. Findings for different prevalence estimates (5% and 15%) are presented in Table 4.

**Table 4:** The number of TP, TN, FM, FP patients by physical examination in 1000 patients

Prevalence	5%	10%	15%
TP	43 (38–48)	86 (76–95)	129 (114–143)
FN	7 (2–12)	14 (5–24)	21 (7–36)
TN	877 (797–939)	831 (755–889)	785 (713–840)
FP	73 (11–153)	69 (11–145)	65 (10–137)

Sensitivity: 0.859 [95% CI 0.761–0.952] at fixed specificity as 0.920

Specificity: 0.923 [95% CI 0.839–0.988] at fixed sensitivity as 0.859

*TP* true positive, *FN* false negative, *TN* true negative, *FP* false positive

The number of patients and 95%CI of TP, FN, TN, FP among the 1000 trauma patients

### Net benefit and decision curve analysis

**Fig. 4:**
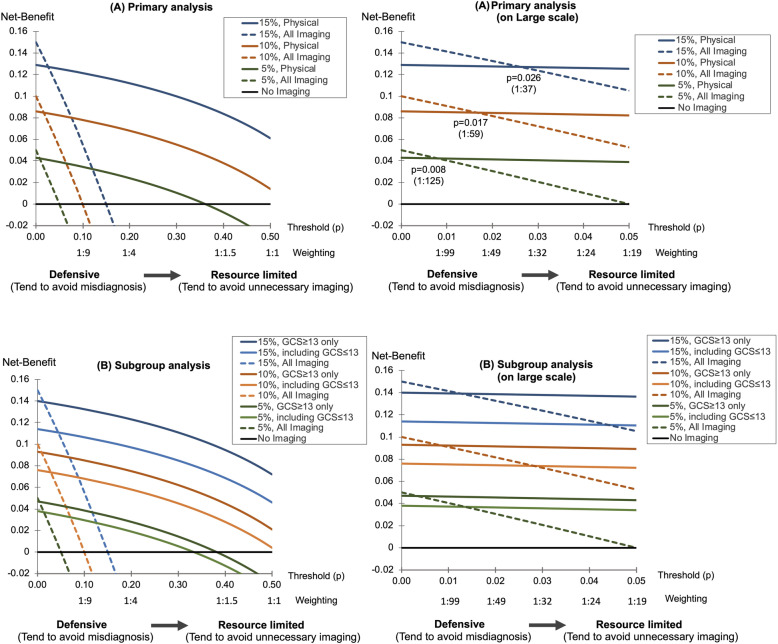
Decision curve analysis of the physical examination. **a** Primary analysis. **b** Subgroup analysis of the level of consciousness, *X*-axis: threshold probability and the weighting, *Y*-axis: net-benefit, lower figure **a**, **b** focusing the threshold range 0–0.05. Colored bold lines: net-benefit of the physical examination under the prevalence 15, 10, 5%; dotted lines: net-benefit by imaging all the patients regardless of physical examination under the prevalence 15, 10, 5%; black bold line: no imaging regardless of physical examination (net-benefit: zero). If the curve of physical examination is under the dotted line of same prevalence, imaging test should be performed in all patients regardless of physical examination. If the curve of physical examination is over the dotted line of same prevalence, imaging test should be performed based on the physical examination findings

Findings from the decision curve analysis at a fixed specificity of 0.92 are shown in Fig. 4a. When the threshold probabilities were set at < 0.008, 0.017, and 0.026 with a 5%, 10%, and 15% prevalence, respectively, the net benefit of imaging was higher than that of physical examination. Otherwise, the net benefit of physical examination was higher than that of any imaging tests.

### Subgroup analysis

**Fig. 5:**
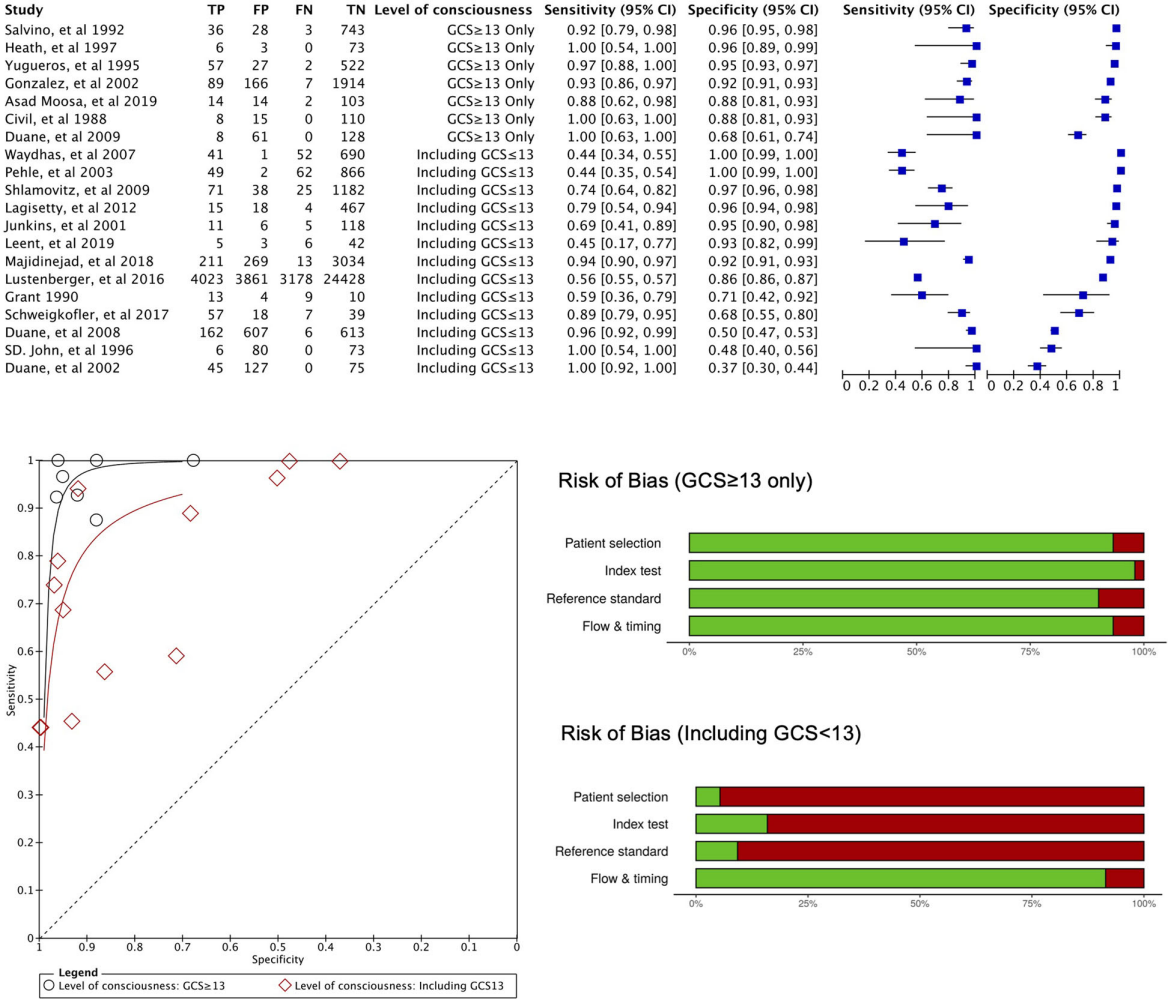
Paired forest plot and HSROC in subgroup analysis (level of consciousness). Risk of bias (GCS ≥ 13 only). Risk of bias (including GCS ≤ 13). Green: low risk of bias or low concern in applicability, red: high risk of bias or high concern in applicability

Although some of the predefined subgroup analysis could not be performed owing to a lack of adequate data or the small number of studies, we were able to assess the heterogeneity of covariates as level of consciousness (GCS ≥ 13 only or including GCS ≤ 13). The level of consciousness subgroup analysis revealed that the overall risk of bias and applicability were respectively low and of low concern in the subgroup without patients who have impaired consciousness compared with the subgroup with patients who have impaired consciousness (Fig. 5 and S-Figure1 in supplementary file). The HSROC parameters captured the between-group heterogeneity (Fig. 5). The DORs were 342.8 (70.8–1659.9) and 43.4 (20.4–92.0) for patients with GCS ≥ 13 (13 studies) and GCS ≤ 13 (7 studies), respectively. The RDOR for subgroup comparisons was 7.9 (1.4–44.6), and the *p* value was 0.027 in the likelihood ratio test. The pooled sensitivity for patients with GCS ≥ 13 based on HSROC parameters was 0.933 (0.847–0.998) at a given specificity of 0.920; for patients with GCS ≤ 13, the corresponding value was 0.761 (0.560–0.932), suggesting that sensitivity among patients with GCS ≥ 13 was higher than that among patients with GCS ≤ 13.

For patients with GCS ≥ 13; threshold probabilities of < 0.003, 0.008, and 0.013; and prevalence of 5%, 10%, and 15%, the net benefit of the imaging tests was higher than that of physical examination (Fig. 4b). Otherwise, the net benefit of physical examination was higher than that of imaging tests. For patients with GCS ≤ 13; threshold probabilities of < 0.014, 0.028, and 0.044; and prevalence of 5%, 10%, and 15%, the net benefit of imaging tests was higher than that of physical examination.

### Sensitivity analysis

In sensitivity analysis, we excluded the following studies to assess the robustness of the results. For the index test, we excluded studies in which the examination type was not relevant to this review, such as neurogenic examination or rectal examination. Furthermore, we excluded studies in which the reference standard was defined as only x-ray or unclear. From post-hoc sensitivity analysis, we excluded studies that were assessed as having high risk of bias in at least one domain. The relevant findings are shown in a supplementary file (S-Figure 2, 3, and 4). Excluding studies with high risk of bias marginally improved diagnostic accuracy, but other exclusions did not affect any estimates.

## Discussion

This systematic review and meta-analysis using the HSROC model revealed that the pooled sensitivity of physical examination for pelvic fracture was 0.859 (0.761–0.952) at a given specificity of 0.92. Furthermore, the pooled sensitivity for trauma patients with GCS ≥ 13 was 0.933 (0.847–0.998), which was higher than that of patients with impaired consciousness (0.761 [0.560–0.932]), at a given specificity of 0.92. Although the overall quality of evidence was low, it became high when studies that included patients with impaired consciousness were excluded. Moreover, decision curve analysis showed that when the threshold probability was < 0.01 and prevalence was 10–15%, imaging tests should be performed for all trauma patients regardless of the physical examination findings. Meanwhile, a threshold probability > 0.05 indicates that physical examination is useful as a screening tool. Overall, the clinical utility of physical examination depends on the prevalence of pelvic fracture, threshold probability, and patients’ consciousness.

### Clinical implication

Imaging tests should be performed for all trauma patients regardless of physical examination findings or patient consciousness status when delivering care at a trauma center or emergency department of a tertiary care center. In general, the clinical utility of a test depends on its diagnostic accuracy, target condition prevalence, patient and physician preference, and physician policy regarding associated risks (misdiagnosis or cost). Therefore, we assumed some scenarios in setting the hypothetical prevalence and policy.

First, we assumed that the prevalence of pelvic fracture was 10–15% at an advanced trauma center in an urban area. Such an institution has access to imaging modalities and implements policies aimed at preventing misdiagnoses, which may increase the risk of a lawsuit. Therefore, the threshold probability was set at 0.01. Under this assumption, decision curve analysis suggested that imaging tests should be performed for all patients regardless of physical examination findings or patients’ level of consciousness. Further, assessment of pelvic ring instability can sometime increase the bleeding by dislocating bones margin [2]. In the situation where the patient is strongly suspected as an unstable pelvic fracture, the net-benefit is subtracted by harm of adverse event; thus, it is also reasonable to perform the imaging test for all patients without physical examination.

Second, we assumed a resource-limited situation, such as in a field hospital at the front lines of war zones or in field triages at the scene of an injury or disaster. In such a condition, setting the threshold probability to 0.05–0.2 is reasonable. Under this assumption, decision curve analysis suggested that physical examination is useful as a screening tool even in cases involving impaired consciousness.

Third, we assumed that pelvic fracture prevalence was 10% at an emergency department of a regional hospital and set the threshold probability at 0.02. In such a situation, decision curve analysis suggested that for patients with a GCS score ≥ 13, physical examination is a useful screening tool; however, for patients with a GCS score ≤ 13, imaging test should be performed. In these scenarios, the clinical utility of physical examination depended on the context; this variability should be considered when making decisions in a clinical setting.

The present findings have implications for further research. First, most studies were set at emergency departments of trauma centers or university hospitals. However, the clinical utility of a physical examination might be higher in a resource-limited environment or at a scene of an injury than in a resource-rich environment. Further studies should evaluate the differences between these settings. Second, although the methodological quality in the subgroup that only included GCS ≥ 13 patients were assessed as having low risk of bias and low concern in applicability, this subgroup was evaluated in other studies as having high risk and high concern because most of these studies excluded patients inappropriately or presented inadequate reports. To ensure a higher quality of evidence, further research is required, particularly studies that include trauma patients with GCS score ≤ 13, are based on rigorous methodology and are transparent in the reporting of their findings.

### Strengths

Previous reviews concluded that physical examination was useful for excluding pelvic fracture in alert trauma patients [7, 9]. By contrast, the present review revealed that the clinical utility of physical examination varied between settings and level of consciousness. In tertiary care settings such as trauma centers, the clinical benefit of physical examination appeared lower than that of imaging tests for all trauma patients. The validity and reliability of the present findings are likely superior to those of previous studies owing to the following reasons.

First, this systematic review was based on a comprehensive literature search. By contrast, two previous systematic reviews of pelvic fracture physical examination [7, 9] failed to incorporate several important studies owing to inadequate search [10, 11, 39, 43, 45, 50]. Second, the current review included study quality assessment and a methodologically rigorous meta-analysis [16–18]. By contrast, two previous systematic reviews had critical limitations to their methodology [7, 9]. One review [7] lacked quality assessment, and both previous reviews followed an unsuitable methodology of meta-analysis that did not include a hierarchical model [7, 9]. Third, in subgroup analysis, we examined between-study heterogeneity in the diagnostic accuracy of patients with and without impaired consciousness; no previous systematic review investigated the sources of heterogeneity. Fourth, we assessed clinical utility by using decision curve analysis; no previous review has assessed the clinical utility of physical examination for pelvic fracture. Given these considerations, this study makes a significant contribution to the literature.

### Limitations

These strengths notwithstanding, this study has some limitations, which should be considered when interpreting its findings. First, there was considerable heterogeneity regarding the patients’ levels of consciousness; studies that included patients with impaired consciousness were of lower quality than those that did not include such patients. Thus, we separated the decision curve analyses and showed that the effect of heterogeneity on the clinical decision was unlikely to be significant. Second, despite a comprehensive search strategy, some relevant studies might have been missed. Third, some of the included studies inadequately reported their findings, thus possibly affecting data extraction and quality assessment. Fourth, the number of studies were limited to 20. If more studies were available, the derived estimates would be more precise, and the sources of heterogeneity could be more adequately explored. Fifth, most included studies were set within the trauma centers or emergency departments of university hospitals; therefore, the generalizability of these findings to other settings is unclear.

## Conclusion

Findings from this review demonstrated that at a threshold probability of < 0.01 and prevalence of 10–15%, imaging tests should be performed for all trauma patients regardless of physical examination findings or patients’ levels of consciousness. However, clinicians should consider the role of physical examination with the prevalence of the target condition and the threshold probability in a given setting.

## Supplementary information

**Additional file 1.** S-Table 1. Excluded literatures by full-text screening. S-Table 2. The detail of the assessment of QUDAS-2 tool. S-Figure 1. The quality of the included studies in the sub-group of level of consciousness. S-Figure 2. The results of sensitivity analysis excluding the studies using index test other than pre-defined in the protocol. S-Figure 3. The results of sensitivity analysis excluding the studies using reference standard as only x-ray or unclear. S-Figure 4. The results of post-hoc sensitivity analysis only excluding the studies evaluated as “High risk of bias”.

## Data Availability

Not applicable.

## Abbreviations

FN: False-negative
TP: True-positive
FP: False-positive
TN: True-negative
CT: Computed tomography
DTA: Diagnostic test accuracy
PRISMA-DTA: Preferred Reporting Items for a Systematic Review and Meta-analysis of Diagnostic Test Accuracy
CIs: Confidence intervals
ROC: Receiver operating curve
HSROC: Hierarchical summary ROC
DOR: Diagnostic odds ratio
RDOR: Relative DOR
GCS: Glasgow Coma Scale
