# Supplementary material for: Diagnostic accuracy of physical examination for detecting pelvic fractures among blunt trauma patients: a systematic review and meta-analysis
Source: World J Emerg Surg. 2020 Oct 2;15:56. doi: 10.1186/s13017-020-00334-z (PMC7531119; doi:10.1186/s13017-020-00334-z)
Supplement: Supplementary file 1 — Additional file 1. S-Table 1. Excluded literatures by full-text screening. S-Table 2. The detail of the assessment of QUDAS-2 tool. S-Figure 1. The quality of the included studies in the sub-group of level of consciousness. S-Figure 2. The results of sensitivity analysis excluding the studies using index test other than pre-defined in the protocol. S-Figure 3. The results of sensitivity analysis excluding the studies using reference standard as only x-ray or unclear. S-Figure 4. The results of post-hoc sensitivity analysis only excluding the studies evaluated as “High risk of bias”. [file 13017_2020_334_MOESM1_ESM.docx]

**Supplementary file**

Diagnostic accuracy of physical examination for detecting pelvic fractures among blunt trauma patients: A systematic review and meta-analysis

**Authors**

Yohei Okada, MD; Norihiro Nishioka, MD; Shigeru Ohtsuru, MD, PhD; Yasushi Tsujimoto, MD, MPH

**Contents**

| Search strategy |
| --- |
| S-Table 1. Excluded literatures by full-text screening |
| S-Table 2. The detail of the assessment of QUDAS-2 tool |
| S-Figure 1. The quality of the included studies in the sub-group of level of consciousness |
| S- Figure 2. The results of sensitivity analysis excluding the studies using index test other than pre-defined in the protocol |
| S- Figure 3. The results of sensitivity analysis excluding the studies using reference standard as only x-ray or unclear |
| S- Figure 4. The results of post-hoc sensitivity analysis only excluding the studies evaluated as “High risk of bias” |
| Reference |

* The review protocol is available on a preprint server (medRexiv)[1] and was prospectively registered with the University Hospital Medical Information Network Clinical Trials Registry (UMIN000038785).[2, 3]

**Search strategy (MEDLINE via Ovid)**

MEDLINE Ovid (Ovid MEDLINE(R) Epub Ahead of Print, In‐Process & Other Non‐Indexed Citations, Ovid MEDLINE(R) Daily and Ovid MEDLINE(R) 1946 to Present)

**Population: Trauma patients**

1: exp "Wounds and Injuries"/OR trauma.ti,ab. OR injur*.ti,ab.

**Index test: Physical examination**

2: exp Physical examination/ OR ((physical or clinical) ADJ2 (diagnosis or sign* OR symptom* OR assessment or finding* OR evaluat* or examination*)).ti,ab,kw

**Target condition: Pelvic fracture**

3: ((fracture* or disrupt* or displac* or injur* or traum* or rupture*) adj2 (pelvi* or ilia* or pubi*)).ti,ab.

**Exclusion:** **Review, case report, and animal studies**

4: Review.pt OR case reports.pt OR (exp animals/ NOT exp humans/)

5: (1 AND 2 AND 3) NOT 4

**Search strategy (EMBASE)**

S1 EMB.EXACT.EXPLODE(Injury)

S2 ab(trauma) OR ti(trauma)

S3 ab(injur*) OR ti(injur*)

S4 S1 OR S2 OR S3

S5 EMB.EXACT.EXPLODE(physical examination)

S6 ab((physical or clinical) N/2 (diagnosis or sign* or symptom* or assessment or finding* or evaluat* or examination*)) OR ti((physical or clinical) N/2 (diagnosis or sign* or symptom* or assessment or finding* or evaluat* or examination*))

S7 ab((fracture* or disrupt* or displac* or injur* or traum* or rupture*) N/2 (pelvi* or ilia* or pubi*)) OR ti((fracture* or disrupt* or displac* or injur* or traum* or rupture*) N/2 (pelvi* or ilia* or pubi*))

S8 S6 OR S5

S9 S8 AND S7 AND S4

**S-Table 1. Excluded literatures by full-text screening**

| **Title** | **Year** | **Author** | **Reason** |
| --- | --- | --- | --- |
| Predictive value of the hip flexion test for fractures of the pelvis | 1996 | Ham, S. J. | Wrong reference |
| The cost-effectiveness of routine pelvic radiography in the evaluation of blunt trauma patients | 1999 | Kaneriya, P. P. | Wrong index test |
| Does ethanol affect the reliability of pelvic bone examination in blunt trauma? | 2000 | Tien, I. Y. | Case-control design |
| Blunt trauma and the role of routine pelvic radiographs | 2001 | Duane, T. M. | Case-control design |
| The clinical presentation of pediatric pelvic fractures | 2001 | Junkins, E. P. | Case-control design |
| The screening pelvic radiograph in pediatric trauma | 2001 | Rees, M. J. | Wrong index test |
| Clinical examination in screening for pelvic fractures in blunt trauma 1 | 2002 | Krantz, Brent E. | Wrong purpose or type of study |
| Pelvic radiography in blunt trauma resuscitation: A diminishing role | 2002 | Guillamondegui, Oscar D. | Wrong index test |
| Physical examination and imaging of hip injuries | 2002 | Kallas, Kerry M. | Wrong reference |
| Clinical effectiveness of the physical examination in diagnosis of posterior pelvic ring injuries | 2003 | McCormick, Joseph P. | Wrong purpose or type of study |
| Validation of a decision instrument to limit pelvic radiography in blunt trauma | 2005 | Gross, Eric Alan | 2x2table unavailable |
| Is routine portable pelvic X-ray in stable multiple trauma patients always justified in a high technology era? | 2007 | Kessel, Boris | Wrong index test |
| Necessity of routine pelvic radiograph in the pediatric blunt trauma patient | 2008 | Ramirez, Dana W. E. | Case-control design |
| Physical Examination is a Poor Screening Test for Abdominal-Pelvic Injury in Adult Blunt Trauma Patients | 2010 | Michetti, Christopher P. | Wrong index test |
| Implementation of a cost-saving algorithm for pelvic radiographs in blunt trauma patients | 2011 | Barleben, Andrew | Wrong index test |
| Analysis of the necessity of routine tests in trauma patients in the emergency department | 2012 | Ozlem Koksal | Wrong index test |
| Indications and performance of pelvic radiography in patients with blunt trauma | 2012 | Holmes, James F. | Wrong index test |
| Role of routine pelvic radiography in initial evaluation of stable, high-energy, blunt trauma patients | 2013 | Paydar, Shahram | Wrong index test |
| Patterns of injury and management of children with pelvic fractures at a non-trauma center | 2014 | Ortega, Henry W. | Wrong index test |
| Clinical and economic effects of selective radiological evaluation of high-energy trauma patients: A prospective experience of a level 1 busy trauma centre | 2015 | Paydar, Shahram | Wrong purpose or type of study |
| Reducing unnecessary portable pelvic radiographs in trauma patients: A resident-driven quality improvement initiative | 2015 | Langer, Jessica M. | Wrong purpose or type of study |
| Significance of clinical examination, CT and MRI scan in the diagnosis of posterior pelvic ring fractures | 2015 | J.V. Nu ̈ chtern | Wrong purpose or type of study |
| Skip and save: Utility of pelvic x-rays in the initial evaluation of blunt trauma patients | 2015 | Soto, Jose Raul | Case-control design |
| Use of the initial trauma CT scan to aid in diagnosis of open pelvic fractures | 2015 | Scolaro, John A. | Wrong index test |
| Pre-hospital pelvic girdle injury: Improving diagnostic accuracy in a physician-led trauma service | 2016 | Yong, E. | Wrong index test |
| Prehospital assessment of injury type and severity in severely injured patients by emergency physicians: An analysis of the Trauma Register DGU | 2017 | Esmer, E. | Wrong index test |
| Negative Stress Examination Under Anesthesia Reliably Predicts Pelvic Ring Union Without Displacement | 2017 | Whiting, Paul S. | Wrong population |
| Straight leg elevation to rule out pelvic injury | 2018 | Bolt, Caroline | Wrong index test |
| The patellar pubic percussion test: a simple bedside tool for suspected occult hip fractures | 2018 | Smeets, Stef Jozef Marie | Wrong purpose or type of study |
| Is there any benefit in the pre-hospital application of pelvic binders in patients with suspected pelvic injuries? | 2019 | Schweigkofler, Uwe | Wrong purpose or type of study |

**S-Table 2. The detail of the assessment of QUDAS-2 tool**

|  |  | **Patients selection** | | | | **Index test** | | | **Ref** | | | **Patients flow** | |
| --- | --- | --- | --- | --- | --- | --- | --- | --- | --- | --- | --- | --- | --- |
| **Author** | **Year** | **Bias** | **Reason** | **Applicability** | **Reason** | **Bias** | **Applicability** | **Reason** | **Bias** | **Applicability** | **Reason** | **Bias** | **Reason** |
| Civil, et al | 1988 | Low |  | Low concern |  | Low | Low concern |  | High | High concern | Blind was unclear | Low |  |
| Grant | 1990 | High | Inappropriate Inclusion | High | Inappropriate Inclusion | Low | Low concern |  | Low | High concern | Only X-ray | High | Inappropriate Exclusion in analysis |
| Salvino, et al | 1992 | Low |  | Low concern |  | Low | Low concern |  | Low | High concern | Only X-ray | Low |  |
| Yugueros, et al | 1995 | Low |  | Low concern |  | Low | Low concern |  | Low | High concern | Only X-ray | Low |  |
| SD. John, et al | 1996 | High | Inappropriate Inclusion | High concern | Inappropriate Inclusion | High | High concern | Index test Positive was unclar | High | High concern | Reader and Blindness | High | Inappropriate Exclusion in analysis |
| Heath, et al | 1997 | High | Inappropriate exclusion | High concern | Inappropriate exclusion | High | High concern | Index test Positive was unclar | High | High concern | Reader and Blindness | High | Inappropriate Exclusion in analysis |
| Junkins, et al | 2000 | Low |  | Low concern |  | Low | Low concern |  | Low | Low concern |  | Low |  |
| Duane, et al | 2002 | High | Inappropriate exclusion | High concern | Inappropriate exclusion | Low | Low concern |  | High | High concern | Reader and Blindness | High | Inappropriate Exclusion in analysis |
| Gonzalez, et al | 2002 | Low |  | Low concern |  | Low | Low concern |  | Low | Low concern |  | Low |  |
| Pehle, et al | 2003 | Low |  | Low concern |  | Low | Low concern |  | High | High concern | Blind was unclear | Low |  |
| Waydhas, et al | 2007 | Low |  | Low concern |  | Low | Low concern |  | Low | Low concern |  | Low |  |
| Duane, et al | 2008 | High | Inappropriate exclusion | High concern | Inappropriate exclusion | Low | Low concern |  | Low | Low concern |  | High | Inappropriate Exclusion in analysis |
| Duane, et al | 2009 | High | Inappropriate exclusion | High concern | Inappropriate exclusion | Low | Low concern |  | High | High concern | Reader and Blindness | High | Inappropriate Exclusion in analysis |
| Shlamovitz, et al | 2009 | High | Inappropriate exclusion | High concern | Inappropriate exclusion | High | High concern | Retrospective | Low | Low concern |  | High | Inappropriate Exclusion in analysis |
| Lagisetty, et al | 2012 | Low |  | Low concern |  | High | High concern | Retrospective | Low | High concern | Only X-ray | High | Inappropriate Exclusion in analysis |
| Lustenberger, et al | 2016 | High | Inappropriate Inclusion | High concern | Inappropriate Inclusion | High | High concern | Index test Positive was unclar | High | High concern | Reader and Blindness | Low |  |
| Majidinejad, et al | 2018 | High | Unclear inclusion criteria | High concern | Unclear inclusion criteria | Low | Low concern |  | High | High concern | Reader and Blindness | Low |  |
| Schweigkofler, et al | 2017 | High | Unclear inclusion criteria | High concern | Unclear inclusion criteria | High | High concern | Index test Positive was unclar | High | High concern | Reader and Blindness | Low |  |
| Leent, et al | 2019 | High | Inappropriate Inclusion | High concern | Inappropriate Inclusion | Low | Low concern |  | High | High concern | Reader and Blindness | High | Inappropriate Exclusion in analysis |
| Moosa, et al | 2019 | Low |  | Low concern |  | Low | Low concern |  | Low | High concern |  | Low |  |

**S-Figure 1. The quality of the included studies in the sub-group of level of consciousness**

**GCS≥13 Only**

**Risk of bias**

**
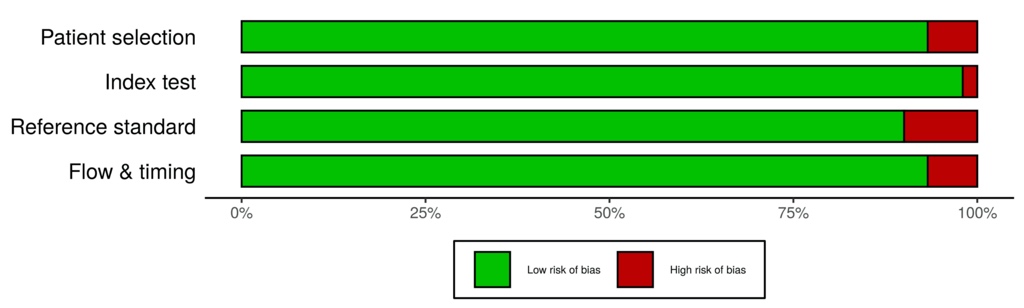
**

**Applicability**

**
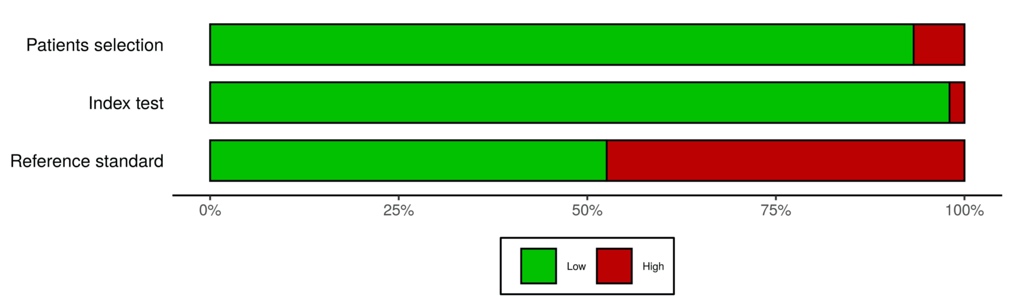
**

**Including GCS≤13**

**Risk of Bias**


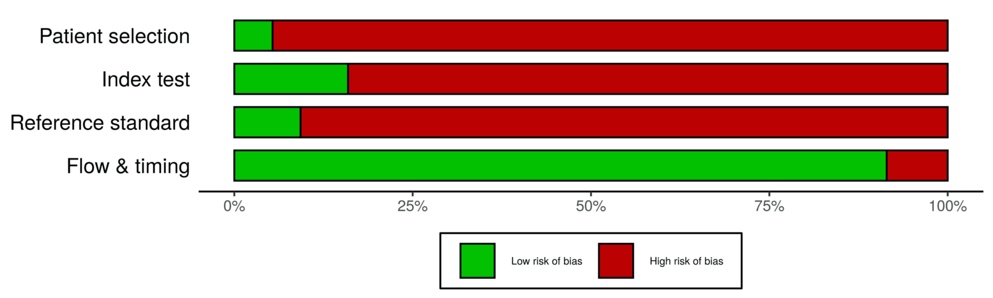


**Applicability**


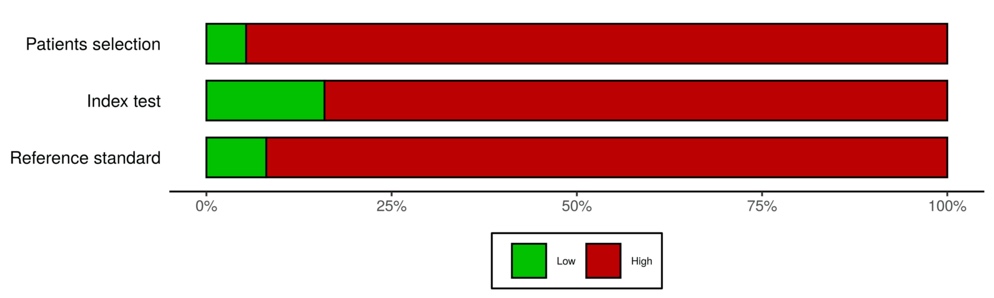


Green: Low risk of bias or low concern in applicability

Red: High risk of bias or high concern in applicability

**S- Figure 2.**

**The results of sensitivity analysis excluding the studies using index test other than pre-defined in the review protocol**

HSROC (Black): Sensitivity analysis only included studies using index test defined in review protocol

HSROC (Gray): Primary analysis included all studies

○: Studies using index test defined in protocol

×: Studies using index test other than pre-defined in review protocol such as neurological examination or rectal examination

DOR in sensitivity analysis: 55.6 [95%CI: 25.2-122.4]

**S-Figure 3.**

**The results of sensitivity analysis excluding the studies using reference standard as only x-ray or unclear**

HSROC (Black): Sensitivity analysis only included studies using reference standard as X-ray or CT

HSROC (Gray): Primary analysis included all studies

○: Studies using reference standard as X-ray or CT

×: Studies using reference standard as X-ray only or unclear

DOR in sensitivity analysis: 72.9 [95%CI: 27.5-193.0]

**S-Figure 4.**

**The results of post-hoc sensitivity analysis only excluding the studies evaluated as “High risk of bias” at least in one domain**

HSROC (Black): Sensitivity analysis only included studies evaluated as “Low risk of bias”

HSROC (Gray): Primary analysis included all studies

○: Studies evaluated as “Low risk of bias”

×: Studies evaluated as “High risk of bias” at least in one domain

DOR in sensitivity analysis: 187.0 [95%CI: 62.0-564.1]

**Reference**

1. Okada Y, Nishioka N, Tsujimoto Y: **Diagnostic accuracy of physical examination to detect the pelvic fractures among the blunt trauma patients; systematic review and meta-analysis**. *medRxiv* 2020:2020.2001.2009.20017129.

2. Booth A, Clarke M, Dooley G, Ghersi D, Moher D, Petticrew M, Stewart L: **The nuts and bolts of PROSPERO: an international prospective register of systematic reviews**. *Systematic reviews* 2012, **1**(1):2.

3. **UMIN Clinical Trials Registry** [<https://www.umin.ac.jp/ctr/index.htm>]
